# Dietary palmitate and oleate differently modulate insulin sensitivity in human skeletal muscle

**DOI:** 10.1007/s00125-021-05596-z

**Published:** 2021-10-26

**Authors:** Theresia Sarabhai, Chrysi Koliaki, Lucia Mastrototaro, Sabine Kahl, Dominik Pesta, Maria Apostolopoulou, Martin Wolkersdorfer, Anna C. Bönner, Pavel Bobrov, Daniel F. Markgraf, Christian Herder, Michael Roden

**Affiliations:** 1 Department of Endocrinology and Diabetology, Medical Faculty and University Hospital Düsseldorf, Heinrich-Heine-University, Düsseldorf, Germany; 2 Institute for Clinical Diabetology, German Diabetes Center, Leibniz Institute for Diabetes Research at Heinrich-Heine-University, Düsseldorf, Germany; 3 German Center for Diabetes Research, Partner Düsseldorf, Neuherberg, Germany; 4 Landesapotheke Salzburg, Department of Production, Hospital Pharmacy, Salzburg, Austria; 5 Institute for Biometrics and Epidemiology, German Diabetes Center, Leibniz Center for Diabetes Research at Heinrich-Heine-University, Düsseldorf, Germany

**Keywords:** Glucose metabolism, Insulin signalling, Lipotoxicity, Monounsaturated fatty acids, Saturated fat, Saturated fat, Skeletal muscle

## Abstract

**Aims/hypothesis:**

Energy-dense nutrition generally induces insulin resistance, but dietary composition may differently affect glucose metabolism. This study investigated initial effects of monounsaturated vs saturated lipid meals on basal and insulin-stimulated myocellular glucose metabolism and insulin signalling.

**Methods:**

In a randomised crossover study, 16 lean metabolically healthy volunteers received single meals containing safflower oil (SAF), palm oil (PAL) or vehicle (VCL). Whole-body glucose metabolism was assessed from glucose disposal (*R*_d_) before and during hyperinsulinaemic–euglycaemic clamps with d-[6,6-^2^H_2_]glucose. In serial skeletal muscle biopsies, subcellular lipid metabolites and insulin signalling were measured before and after meals.

**Results:**

SAF and PAL raised plasma oleate, but only PAL significantly increased plasma palmitate concentrations. SAF and PAL increased myocellular diacylglycerol and activated protein kinase C (PKC) isoform θ (*p* < 0.05) but only PAL activated PKCɛ. Moreover, PAL led to increased myocellular ceramides along with stimulated PKCζ translocation (*p* < 0.05 vs SAF). During clamp, SAF and PAL both decreased insulin-stimulated *R*_d_ (*p* < 0.05 vs VCL), but non-oxidative glucose disposal was lower after PAL compared with SAF (*p* < 0.05). Muscle serine^1101^-phosphorylation of IRS-1 was increased upon SAF and PAL consumption (*p* < 0.05), whereas PAL decreased serine^473^-phosphorylation of Akt more than SAF (*p* < 0.05).

**Conclusions/interpretation:**

Lipid-induced myocellular insulin resistance is likely more pronounced with palmitate than with oleate and is associated with PKC isoforms activation and inhibitory insulin signalling.

**Trial registration:**

ClinicalTrials.gov.NCT01736202.

**Funding:**

German Federal Ministry of Health, Ministry of Culture and Science of the State North Rhine-Westphalia, German Federal Ministry of Education and Research, European Regional Development Fund, German Research Foundation, German Center for Diabetes Research.

**Graphical abstract:**

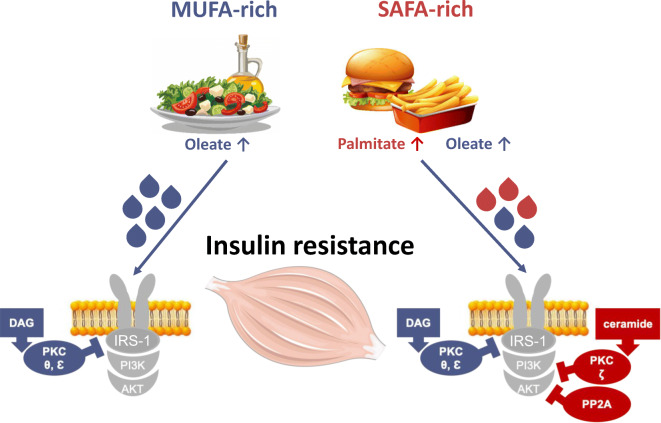

**Supplementary Information:**

The online version contains peer-reviewed but unedited supplementary material available at 10.1007/s00125-021-05596-z.

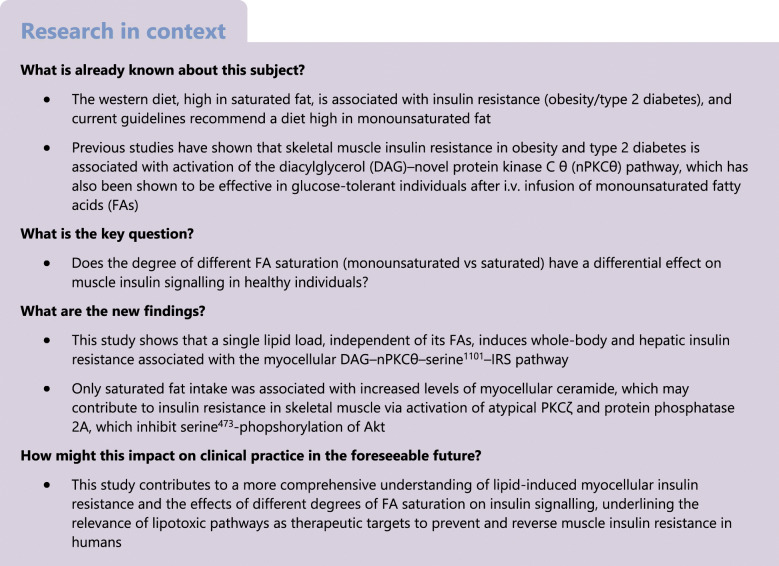



## Introduction

Western-style diets, as defined by high intake of energy and fat, have been related to the rising prevalence of insulin-resistant states such as obesity and type 2 diabetes [1, 2]. Although current guidelines recommend diets low in saturated fatty acid (FA) for type 2 diabetes and CVD, the evidence remains limited [3, 4].

Previously, we have demonstrated that the skeletal muscle insulin resistance in obesity and type 2 diabetes associates with activation of the myocellular diacylglycerol (DAG)–novel protein kinase C (nPKC) isoform θ pathway, an inhibitory cascade of proximal insulin signalling [5, 6]. In line with results from preclinical models [7], we showed that this pathway is also operative in glucose-tolerant humans upon i.v. infusion of lipid emulsions containing predominately monounsaturated FA, as well as in obese individuals and those with type 2 diabetes without any infusion [6]. Of note, this study did not detect changes in other mechanisms that have been postulated to induce insulin resistance, such as sphingolipid mediators, abnormal mitochondrial function or low-grade inflammation, in some [8] but not all previous human studies [6, 9, 10]. Among other causes, different outcomes may result from the degree of NEFA saturation, which can differently affect metabolism and risk of type 2 diabetes and CVD, as saturated FA, but not monounsaturated FA, are considered harmful [11, 12]. Monounsaturated FA availability is known to activate the DAG–nPKCθ pathway [6], whereas saturated FA can increase intracellular ceramides and stimulate protein phosphatase 2A (PP2A) and the atypical protein kinase C (aPKC) isoform ζ, which inhibit activation of Akt [1]. On the other hand, oleate may even protect against palmitate-induced insulin resistance, as demonstrated in L6 myotubes [13]. However, the temporal sequence of the molecular events in human skeletal muscle upon ingestion of differently composed lipid meals remains unknown.

**Fig. 1:**
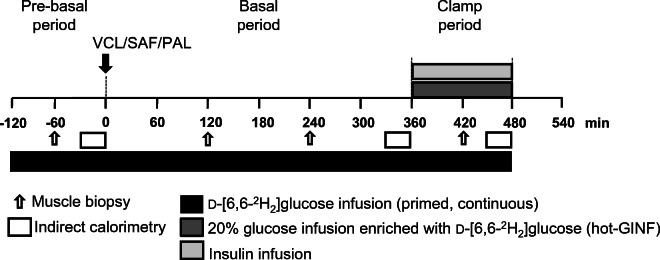
Study design. Lean, healthy adults (10 male and 6 female) randomly ingested either one dose of PAL, SAF or VCL (water) at time point 0 min on three occasions during a period of 12 weeks. Starting at −120 min, d-[6,6-^2^H_2_]glucose was infused up to +480 min. Muscle biopsies were taken at time points −60 min, +120 min, +240 min and +420 min. From +360 min to +480 min, a hyperinsulinaemic–euglycaemic clamp test was performed according to the ‘hot’ glucose infusion (hot-GINF) protocol

We aimed to compare the acute effects of saturated fat (palm oil [PAL]) and monounsaturated fat (safflower oil [SAF]) with water (vehicle [VCL]) on the following variables in young, lean and metabolically healthy humans (ESM Table 1): (1) whole-body insulin sensitivity; (2) (sub)cellular distribution of lipid intermediates; (3) insulin signalling; and (4) mitochondrial oxidative capacity in skeletal muscle. Thus, we performed comprehensive metabolic phenotyping using serial biopsies before and after 2 h, 4 h, as well as 7 h of the respective interventions (Fig. 1 and ESM Fig. 1).

## Methods

### Volunteers

The study was registered at Clinicaltrials.gov (registration no. NCT01736202), approved by the ethics board of Heinrich Heine University of Düsseldorf (reference 3107) and performed according to the Declaration of Helsinki, 2013. All participants (ten men, six women) gave their written informed consent prior to enrolment in this randomised, placebo-controlled, crossover trial. Inclusion criteria were age of 20–40 years and BMI of 20–25 kg/m^2^. Exclusion criteria were family history of diabetes, dysglycaemia, menstrual irregularities, history of smoking, alcohol or drug abuse and other acute or chronic diseases including cancer, as well as any medication intake affecting insulin sensitivity, immune system or lipid metabolism. All volunteers had a screening visit for medical history and clinical examination, lean body mass assessment, routine laboratory tests and a 75 g OGTT. Eligible participants were instructed to maintain and record a carbohydrate-rich diet and to avoid intense physical activity for 3 days prior to all study days. Female participants were examined only between days 5 and 9 of their menstrual cycle. All participants were randomly assigned to the three study days, spaced by an interval of 3 weeks (Fig. 1).

### Randomisation

The random allocation sequence (1:1) was generated by an expert statistician (PB) using SAS software, version 9.3. (SAS Institute, Cary, NC, USA). Participants were randomly assigned to their treatment order by an independent person not involved in the study at the German Diabetes Center (DDZ). The randomisation list was kept by this person and was not accessible to the study personnel. Study participants, medical staff and researchers were blinded until completion of the study.

### Experimental protocol

The study day comprised three periods (Fig. 1): pre-basal (−120 min to 0 min); basal (0 min to +360 min); and clamp (+360 min to +480 min). All participants arrived at DDZ, after a 10 h overnight fast at 07:30 h (−120 min; Fig. 1). Two i.v. catheters were inserted into contralateral forearm veins. A continuous infusion (0.036 mg [kg body weight (BW)]^−1^ min^−1^) of d-[6,6-^2^H_2_]glucose (99% enrichment; Cambridge Isotope Laboratories, Andover, MA, USA) was given from −120 min to +480 min [11], following a 10-min priming bolus (0.36 mg [kg BW]^−1^ min^−1^ [mg/dl fasting blood glucose]). At 0 min, participants received one of three interventions: (1) PAL ~1.18 g/kg BW (48% saturated FA, 35% monounsaturated FA, 15% polyunsaturated FA; Biopalm, Landkrone, Hamburg, Germany); (2) SAF ~1.18 g/kg BW (8% saturated FA, 65% monounsaturated FA, 23% polyunsaturated FA; Mazola, Elmshorn, Germany); or (3) control (VCL) ~1.18 ml/kg BW of bottled still water (Ahrtal Quelle, Sinzig, Germany). Participants drank 92 g (if BW >70 kg) or 80 g (if BW <70 kg) of the lipid drinks (heated up to 60°C, mixed with 1.84 g or 1.60 g emulsifier [Glice; Texturas, Albert y Ferran Adria, Barcelona, Spain], 9 g or 8 g sugar-free vanilla syrup [Torani, San Francisco, CA, USA] and 81.2 ml or 70.4 ml bottled still water, respectively. The VCL drink contained 173.2 ml or 150.4 ml still water. Participant and investigator were blinded to treatment sequence. Each intervention (0 min) was followed by a hyperinsulinaemic–euglycaemic clamp: a bolus of 80 mU m^−2^ (body surface area [BSA]) min^−1^ for 8 min, followed by continuous infusion of 40 mU m^−2^ (BSA) min^−1^ of human short-acting insulin (Insuman Rapid; Sanofi, Frankfurt, Germany) from +360 min to +480 min. Blood glucose was maintained at 5 mmol/l by adapting rates of the 20% glucose infusion (Braun, Melsungen, Germany), enriched with d-[6,6-^2^H_2_]glucose (ESM Fig. 2). Further blood samples were collected at timed intervals [6]. Vital function (heart rate, BP, body temperature) was monitored every 60 min.

### Indirect calorimetry

Indirect calorimetry was performed in the canopy mode using Vmax Encore 29n (CareFusion, Höchberg, Germany) during the last 30 min of the pre-basal, basal and clamp periods [5, 11].

### Skeletal muscle biopsy

Biopsies were taken at −60, +120, +240 and + 420 min from the vastus lateralis muscle of both legs. Under local anaesthesia, muscle specimens were obtained by a modified Bergström needle with suction and were immediately blotted free of extramyocellular tissue or blood, frozen in liquid nitrogen, weighed and stored at −80°C [5].

### Plasma FA analysis

NEFA were analysed as their FA methyl esters (FAMEs) using GC–MS, as described in detail elsewhere [10, 14]. Direct transesterification of all classes of lipids was carried out in a one-step reaction [14]. In brief, lipids were extracted from plasma after addition of internal standard (heptadecanoic acid) using isopropyl alcohol–heptane–sulfuric acid (40:10:1) (Merck, Darmstadt, Germany) and 0.01% butylated hydroxytoluene (≥99%, B1378; Sigma-Aldrich, St Louis, MO, USA). Lipids were separated by thin-layer chromatography using heptane–diethylether–acetic acid (80:30:1) (Merck) as mobile phase. FAs were extracted from silica gel in benzol–methanol (1:4) (Merck) overnight and derivatised to their corresponding methyl esters by addition of acetyl chloride (Merck) and incubation at 100°C for 1 h. After addition of benzol (Merck) and centrifugation, the FAME-containing supernatant fraction was analysed on a Hewlett Packard 6890 gas chromatograph (Palo Alto, CA, USA) interfaced to a Hewlett Packard 5975 mass selective detector. Calibration curves of reference FAs were processed in parallel for quantification [10].

### Myocellular lipids

For quantification of lipid metabolites in subcellular compartments, lipids were extracted, purified and analysed from frozen tissue samples, using liquid chromatography tandem-mass spectrometry (LC-MS/MS) as previously described [5, 15].

### Myocellular signalling

Insulin signalling was assessed by western blotting. Total soluble proteins were extracted from approximately 30 mg of frozen skeletal muscle and homogenised in 300 μl of lysis buffer (25 mol/l Tris-HCl, 1 mmol/l EDTA, 150 mmol/l NaCl, 0.20% NP-40) with protease (cOmplete Tablets, EASYpack; Roche Diagnostics, Basel, Switzerland) and phosphatase (PhosSTOP, EASYpack; Roche Diagnostics) inhibitors. Activities of PKCs were assessed from the ratios of the protein contents in membrane and cytosol fractions upon differential centrifugation. A total of 50 mg of frozen muscle tissue was homogenised in 300 μl of lysis buffer A (25 mmol/l Tris-HCL, 1 mmol/l EDTA, 150 mmol/l NaCl, 0.20% NP-40, with protease and phosphatase inhibitors), centrifuged (100,000 *g*, 1 h at 4°C), and the supernatant fraction containing the cytosolic fraction was transferred into a fresh tube, while the pellet was dissolved in 110 μl of buffer B (250 mmol/l Tris-HCL, 1 mmol/l EDTA, 0.25 mmol/l EGTA, 2% Triton X-100) using a homogeniser. A second centrifugation step (100,000 *g*, 1 h at 4°C) was performed, and the supernatant (membrane fraction) was collected [16]. Proteins concentrations were determined using the BCA (bicinchoninic acid) Assay Kit (Thermo Fisher Scientific, Waltham, MA, USA). Aliquots of 30 μg of total proteins, as well as cytosolic and membrane fractions, were loaded onto an SDS–polyacrylamide gradient gel (4–20% Mini-PROTEAN TGX Precast Protein Gels 190; Bio-Rad, CA, USA) and electrophoresed. After blocking, the membranes were incubated with primary antibodies diluted 1:1000, if not differently specified, in combination with the respective horseradish peroxidase (HRP)-conjugated secondary anti-rabbit antibody, diluted 1:2500, or anti-mouse, diluted 1:1000. Primary antibodies, purchased from Cell Signaling Technology (Danvers, MA, USA), were as follows: Akt (9272); serine^473^-phosphorylation of Akt (9271); serine^1101^-phosphorylation of IRS-1 (2385); stress-activated protein kinase (SAPK)/ c-Jun N-terminal kinase (JNK) (9252); threonine^183^-phosphorylation and thyrosine^185^-phosphorylation of SAPK/JNK (9251); PP2A subunit (2041); aPKCζ (9372); and GAPDH (2118; 1:5000) as housekeeping protein for the soluble and cytosolic fractions. nPKCθ (610090) and nPKCε (610086) were obtained from BD Biosciences, IRS-1 (06–248) from Millipore, and Na^+^/K^+^-ATPase, as housekeeping protein for membrane fractions, from Abcam (Ab76020; 1:10,000). Proteins were detected using a Bio-Rad ChemiDoc MP Imaging System in combination with ImageLab 6.0.1 software (Bio-Rad 199 Laboratories) for densitometric analysis. Data are expressed in arbitrary units and normalised to housekeeping protein.

### Mitochondrial content and function

Mitochondrial respiration was assessed by high-resolution respirometry (Oxygraph 2 k, O2k; Oroboros instruments, Innsbruck, Austria) in freshly harvested permeabilised muscle fibres after applying sequential substrate–uncoupler–inhibitor protocols as described elsewhere [17]. H_2_O_2_ emission from permeabilised muscle fibres was quantified by high-resolution respirometry with Amplex Red as previously described [17]. Citrate synthase activity (CSA) was measured spectrophotometrically (CSA Kit; Sigma-Aldrich) as a surrogate marker for mitochondrial content. Mitochondrial respiration and H_2_O_2_ emission were normalised to individual CSA values to account for differences in mitochondrial content.

### Circulating metabolites and hormones

Plasma concentrations of insulin, gastric inhibitory polypeptide (GIP), glucagon-like peptide-1 (GLP-1), glycerol, glucagon, NEFA, cortisol, alanine aminotransferase (ALT), aspartate aminotransferase (AST), triacylglycerols and chylomicrons as well as blood concentration of glucose and HbA_1c_ were measured as described elsewhere [11]. Plasma glycerol was measured enzymatically (r-Biopharm, Darmstadt, Germany). Serum concentrations of IL-6 and TNF-α were quantified using the respective Quantikine HS ELISA kits (R&D Systems/BioTechne, Wiesbaden, Germany) [11].

### ^2^H-labelled glucose

Measurement of blood [^2^H_2_]glucose atom percent enrichment was performed on a Hewlett Packard 6890 gas chromatograph equipped with a 25 m CPSil5CB capillary column (0.2 mm i. d., 0.12 μm film thickness; Chrompack/Varian, Middelburg, the Netherlands) and interfaced to a Hewlett Packard 5975 mass selective detector [11].

### Calculations

During basal and clamp periods, whole-body rate of glucose disposal (*R*_d_) was calculated from [^2^H_2_]glucose enrichments (Steele’s steady-state equation). During the basal period, *R*_d_ was given as *R*_d_ divided by the mean plasma insulin levels of the last 30 min of the respective period (+330 min to +360 min) [10]. Endogenous glucose production (EGP) was calculated from the rate of appearance (*R*_a_) and was expressed as EGP × insulin levels of the last 30 min of basal period (+330 min to +360 min) [18]. During the steady-state clamp period (+450 min to +480 min), EGP suppression (in %) was calculated to estimate hepatic insulin sensitivity [10]. Incremental areas under the curve (iAUCs) were calculated (basal and clamp period combined) using the trapezoidal rule corrected for the respective AUC [10].

### Statistics

The power calculation is based on a previous study on oral lipid-induced insulin resistance, using two simultaneous two-sided paired *t* tests, resulting in a sample size of *n* = 16 with a multiplicity adjusted α of 0.025 and a power of 85% [5]. Results are presented as means ± SEM (figures), means ± SD for normally distributed variables or median with IQR (first to third quartile) for log normally distributed variables (ESM Table 1) and compared by mixed model repeated measures ANOVA adjusted to BMI, age and sex and with Tukey–Kramer correction. Comparison of changes within one participant was done using a two-side paired *t* test. Variables with skewed distributions were log_*e*_-transformed before analysis. Statistical significance of differences was defined at *p* < 0.05. Calculations were performed using SAS version 9.4 (SAS Institute).

## Results

### PAL and SAF similarly increase plasma chylomicrons but only PAL raises palmitic acid concentrations

**Fig. 2:**
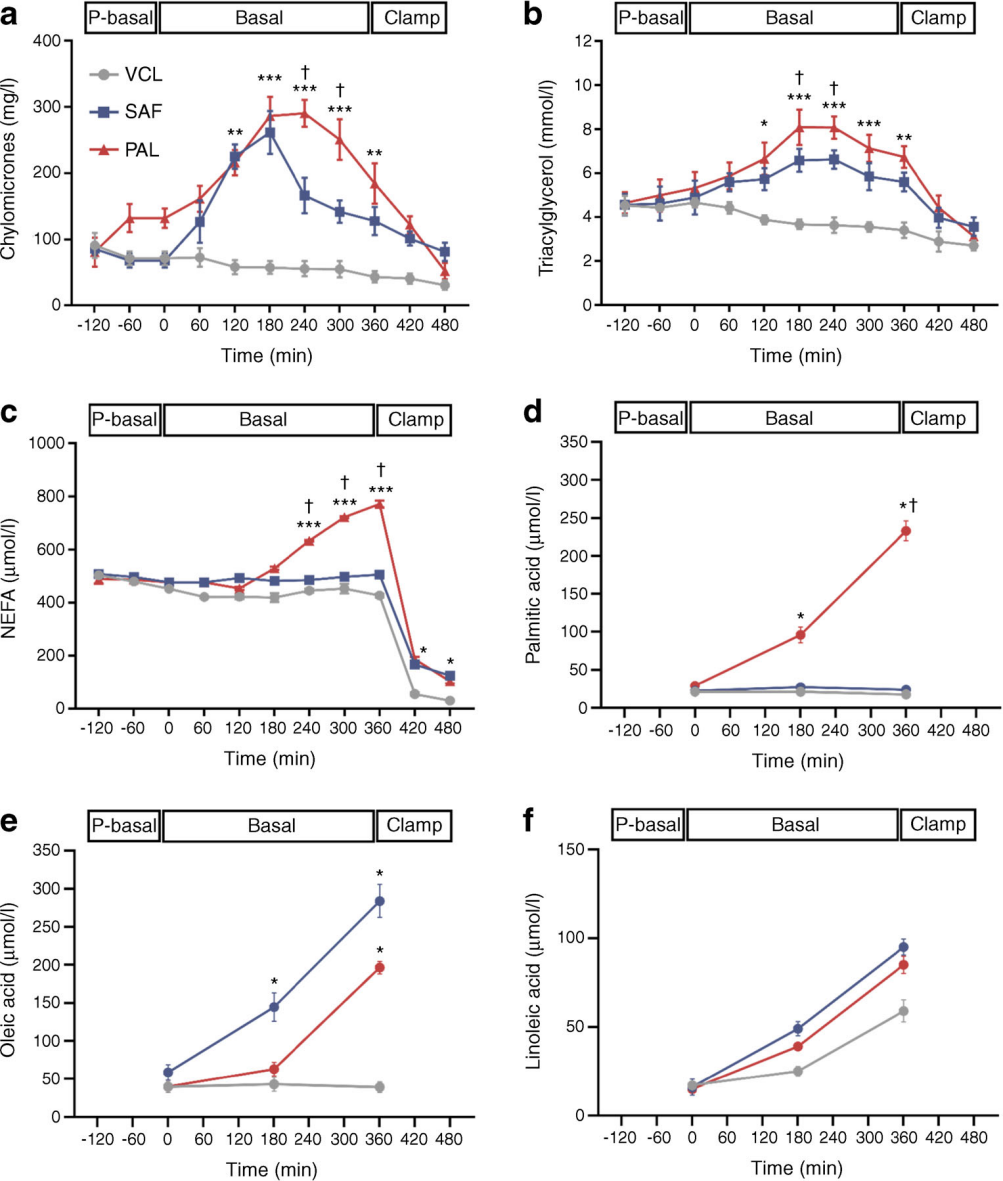
Time courses of circulating lipid metabolites in healthy humans. Plasma concentrations of chylomicrons (**a**), triacylglycerol (**b**), total NEFA (**c**), palmitic acid (**d**), oleic acid (**e**) and linoleic acid (**f**) after ingestion of PAL (red), SAF (blue) or VCL (water, grey) at 0 min. Data are shown as means ± SEM; *n* = 16 (chylomicrons *n* = 10). *p < 0.05, **p < 0.01 and ***p < 0.001 vs VCL at same time point; ^†^*p* < 0.05 for PAL vs SAF at same time point (ANOVA adjusted for repeated measures with Tukey–Kramer correction for each time point between interventions). P-basal, pre-basal

Time-dependent changes were analysed by comparing the iAUC. After both interventions, plasma chylomicrons similarly increased by ~65% from the pre-basal period (*p* = 0.006 for PAL vs VCL; *p* = 0.010 for SAF vs VCL; Fig. 2a). Likewise, plasma triacylglycerols increased by ~37% and ~28% after PAL and SAF, respectively (*p* = 0.004 for PAL vs VCL; *p* = 0.002 for SAF vs VCL; Fig. 2b). Plasma total NEFA were 13% and 24% higher after PAL compared with SAF or VCL, respectively (*p* = 0.0008 and *p* = 0.0005; Fig. 2c). During the basal period, after PAL, plasma palmitic acid was ~80% higher than after SAF or VCL (*p* = 0.0010 and *p* = 0.0005, respectively; Fig. 2d). Plasma oleic acid increased by ~75% after SAF and by ~54% after PAL, compared with VCL (*p* = 0.022 and *p* = 0.042, respectively; Fig. 2e). Plasma linoleic acid rose by ~82% after SAF and by ~78% after PAL, compared with VCL (*p* = 0.001 and *p* = 0.036, respectively; Fig. 2f). There were a few differences for other NEFA species at certain time points, but the respective iAUCs for the PAL and SAF interventions were comparable (ESM Table 2).

### PAL and SAF raise plasma glucagon and GIP but only SAF raises plasma GLP-1

**Fig. 3:**
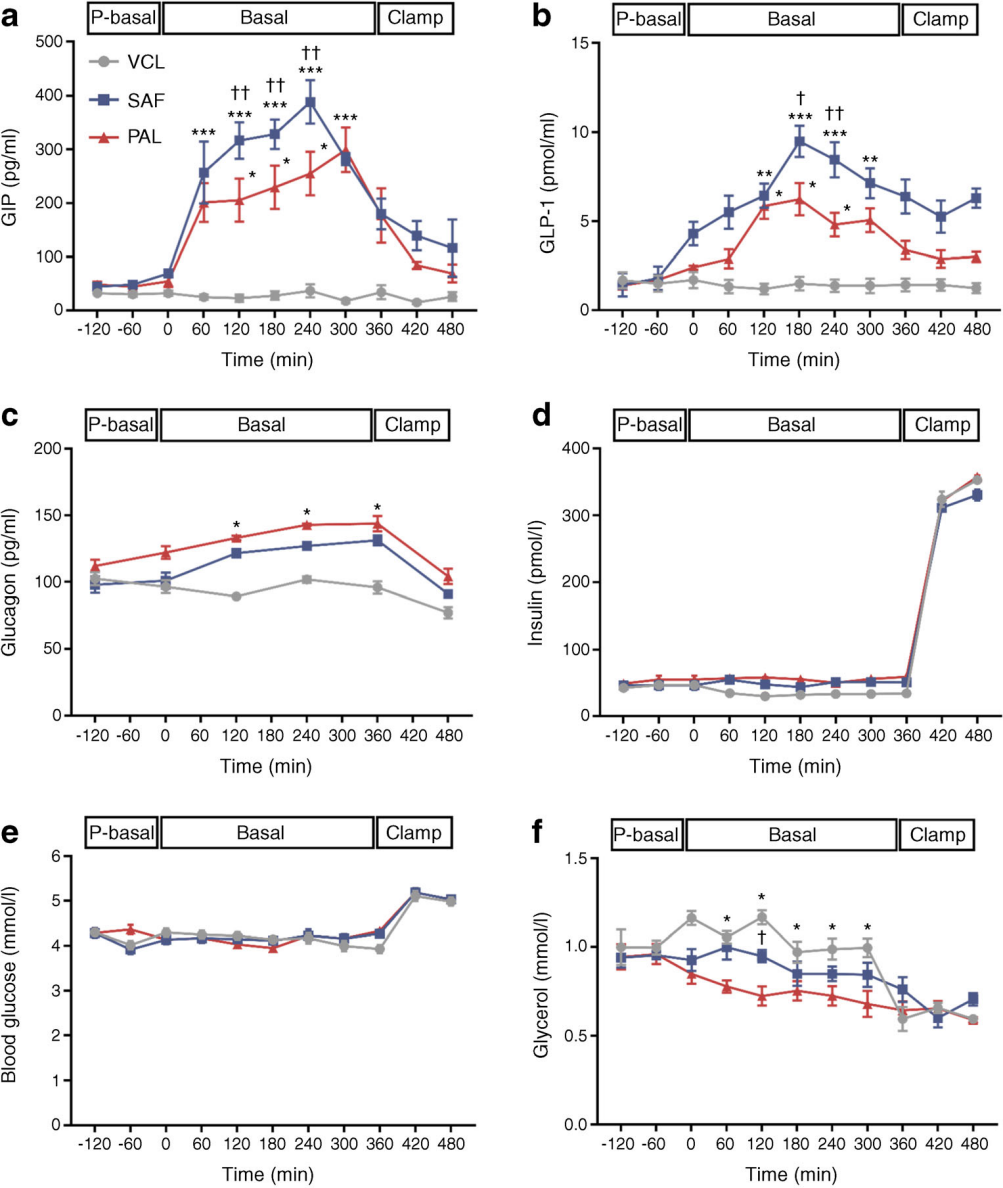
Time courses of circulating hormones and metabolites in healthy humans. Concentrations of plasma GIP (**a**), plasma GLP-1 (**b**), plasma glucagon (**c**), plasma insulin (**d**), blood glucose (**e**) and plasma glycerol (**f**) are presented after ingestion of PAL (red), SAF (blue) or VCL (water, grey) at 0 min. Data are shown as means ± SEM; insulin and blood glucose *n* = 16; GIP, GLP-1, glucagon and glycerol, *n* = 4. *p < 0.05, **p < 0.01 and ***p < 0.001 vs VCL at same time point; ^†^*p* < 0.05 and ^††^*p* < 0.01 for PAL vs SAF at same time point (ANOVA adjusted for repeated measures with Tukey–Kramer correction for each time point between interventions). P-basal, pre-basal

Time-dependent changes were analysed by comparing the iAUC of the respective parameters. From the pre-basal period, plasma GIP increased eightfold and fivefold after SAF and PAL, respectively (*p* = 0.009 and *p* = 0.012 vs VCL; Fig. 3a). After SAF, plasma GLP-1 increased by sixfold and 2.5-fold compared with VCL and PAL, respectively (*p* = 0.001 and *p* = 0.039; *p* = 0.071 for PAL vs VCL; Fig. 3b). During the basal period, plasma glucagon was ~25% and ~20% higher after PAL and SAF, respectively, compared with VCL (*p* = 0.021 and *p* = 0.044; Fig. 3c). Plasma insulin and blood glucose did not differ between the interventions (Fig. 3d,e). Plasma glycerol decreased (by ~30% from pre-basal period), only after PAL, compared with VCL (*p* = 0.041; Fig. 3f).

### PAL and SAF lead to whole-body insulin resistance during postprandial hyperinsulinaemia

**Fig. 4:**
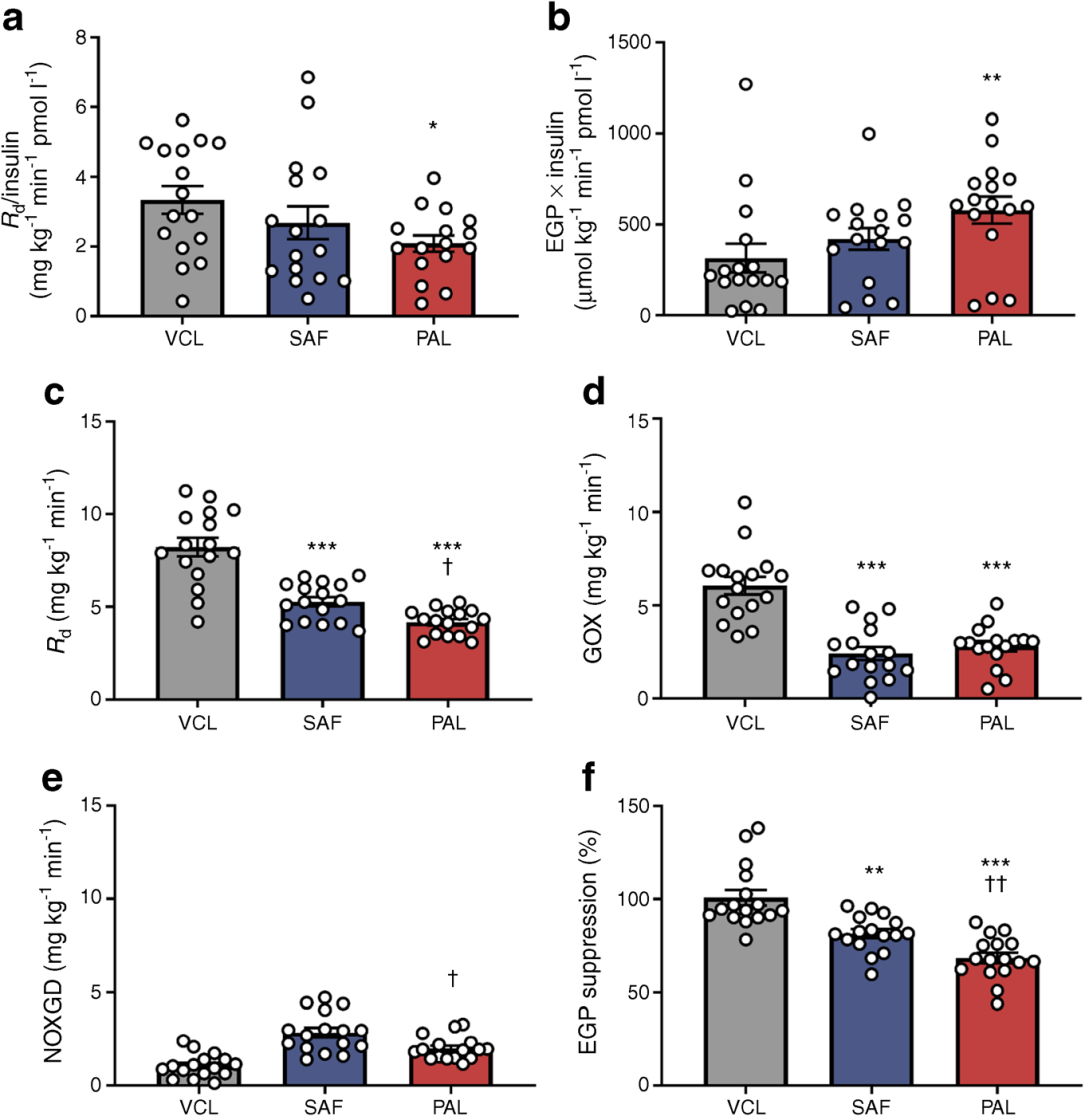
Rates of whole-body glucose disposal (*R*_d_) and EGP during basal and clamp periods in healthy humans. (**a**, **b**) During the last 30 min of basal period (+330 min to +360 min), rates of glucose metabolism are presented in the context of the ambient plasma insulin concentration: *R*_d_/insulin (**a**) and EGP × insulin (**b**). (**c**–**f**) During clamp steady-state (+450 min to +480 min), insulin-stimulated *R*_d_ (**c**), rate of GOX (**d**), rate of NOXGD (**e**) and EGP suppression (**f**) are presented after PAL (red), SAF (blue) or VCL (water, grey) ingestion at 0 min. Data are shown as means ± SEM; *n* = 16. *p < 0.05, **p < 0.01 and ***p < 0.001 vs VCL; ^†^*p* < 0.05 and ^††^*p* < 0.01 for PAL vs SAF (ANOVA adjusted for repeated measures with Tukey–Kramer correction)

During the basal period, whole-body resting energy expenditure (REE) (PAL 7317 ± 309 kJ/day; SAF 7606 ± 267 kJ/day; VCL 7296 ± 259 kJ/day), rates of lipid oxidation (LOX) (PAL 1.0 ± 0.1 mg kg^−1^ min^−1^; SAF 1.1 ± 0.2 mg kg^−1^ min^−1^; VCL 1.1 ± 0.1 mg kg^−1^ min^−1^) and glucose oxidation (GOX) (PAL 2.2 ± 0.2 mg kg^−1^ min^−1^; SAF 2.1 ± 0.5 mg kg^−1^ min^−1^; VCL 2.1 ± 0.2 mg kg^−1^ min^−1^) were not different between the interventions. Only after PAL, whole-body glucose disappearance (*R*_d_/insulin) was 28% lower (*p* = 0.036; Fig. 4a) and EGP × insulin was 38% higher compared with VCL (*p* = 0.009; Fig. 4b).

During the steady-state of the clamp, reflecting postprandial hyperinsulinaemia (Fig. 3d and ESM Table 3), REE was also comparable between the interventions (PAL 7556 ± 330 kJ/day; SAF 7543 ± 305 kJ/day; VCL 7368 ± 284 kJ/day). After PAL and SAF, LOX was 85% and 70% higher compared with VCL, respectively (PAL 3.4 ± 0.9 mg kg^−1^ min^−1^ [*p* = 0.013 vs VCL]; SAF 2.0 ± 0.7 mg kg^−1^ min^−1^ [*p* = 0.019 vs VCL]; VCL 0.5 ± 0.2 mg kg^−1^ min^−1^). Insulin-stimulated *R*_d_ was 49% and 36% lower after PAL and SAF, respectively, compared with VCL (*p* < 0.0001 and *p* = 0.0002; Fig. 4c), partly due to 69% and 76% lower GOX after PAL and SAF (*p* = 0.003 and *p* = 0.004 vs VCL, respectively; Fig. 4d). Further, insulin-stimulated *R*_d_ was 13% lower after PAL than after SAF (*p* = 0.041), due to 59% lower non-oxidative glucose disposal (NOXGD) (*p* = 0.008 vs SAF; Fig. 4e). Hepatic insulin resistance was 39% and 24% lower after PAL and SAF (*p* < 0.0001 and *p* = 0.0010 vs VCL, respectively; Fig. 4f). No sex differences were found between interventions (ESM Fig. 3).

### PAL and SAF associate with the DAG–nPKC pathway

**Fig. 5:**
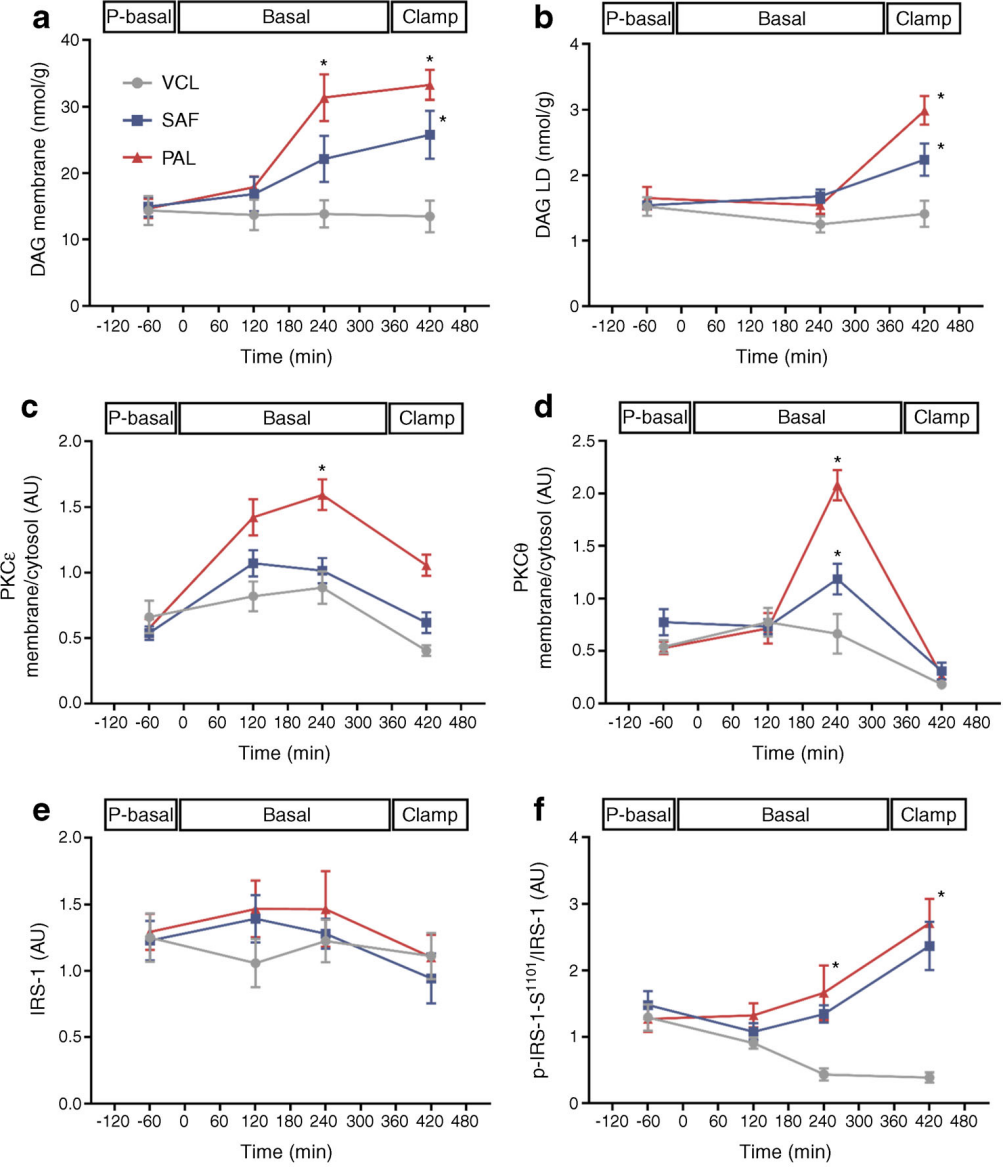
Myocellular lipid metabolites and insulin signalling (DAG–nPKC pathway) in healthy humans. DAG species 18–1:18–1, 16–0:16–0 and 16–0:18–1 in the cell membrane fraction (**a**), DAG species 18–1:18–1, 16–0:16–0 and 16–0:18–1 in the lipid droplet fraction (**b**), nPKCε activation (**c**), nPKCθ activation (**d**) and IRS-1 levels (**e**) as well as serine^1101^-phosphorylation of IRS-1 relative to IRS-1 (**f**) during the pre-basal, basal and clamp periods after ingestion of PAL (red), SAF (blue) or VCL (water, grey) at 0 min. Expression signals on immunoblots are expressed in arbitrary units (AU) after normalising against GAPDH for total and cytosolic proteins and against Na^+^/K^+^-ATPase for membrane proteins. Data are shown as means ± SEM; *n* = 16 at time point −60 min, *n* = 10 at +120 min, *n* = 6 at +240 min and +420 min. *p < 0.05 vs VCL at same time point (ANOVA adjusted for repeated measures with Tukey–Kramer correction for each time point between interventions). LD, lipid droplet fraction; P-basal, pre-basal

Time-dependent changes were analysed by comparing the iAUC of the respective parameters. Across basal and clamp periods combined, the concentration of 18–1:18–1; 16–0:16–0; 16–0:18–1 DAG species in the membrane compartment was increased by ~43% (*p* = 0.032) after PAL and by ~30% (*p* = 0.041) after SAF (Fig. 5a). The accumulation of these DAG species in lipid droplets increased by ~25% after PAL only (*p* = 0.034 vs VCL; *p* = 0.122 vs SAF; Fig. 5b). Membrane translocation of nPKCε increased by 75% after PAL (*p* = 0.003 vs VCL; *p* < 0.0001 vs SAF; Fig. 5c) and tended to rise after SAF but did not reach statistical significance (*p* = 0.061 vs VCL). Activation of nPKCθ was 94% higher with PAL (*p* = 0.004 vs VCL; *p* = 0.009 vs SAF) and 31% higher with SAF (*p* = 0.041 vs VCL; Fig. 5d). Myocellular serine^1101^-phosphorylation of IRS-1 was increased by 57% and 52% upon PAL and SAF ingestion (*p* = 0.037 and *p* = 0.039, respectively, vs VCL; Fig. 5e, f).

### PAL further associates with the ceramide–aPKCζ–PP2A pathway

**Fig. 6:**
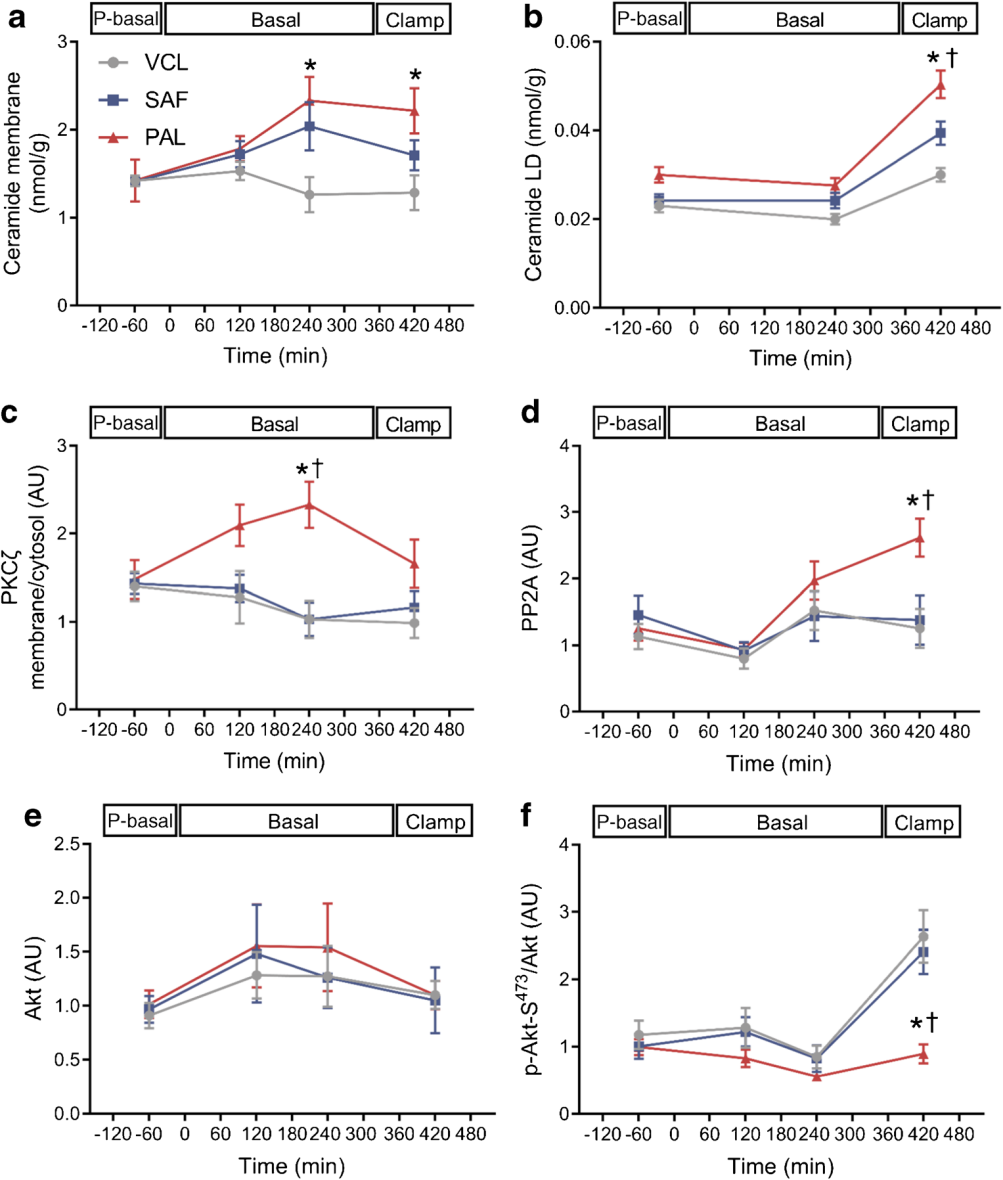
Myocellular lipid metabolites and insulin signalling (ceramide–aPKC pathway) in healthy humans. Ceramide species 16:0, 18:0 and 18:1 in the cell membrane fraction (**a**), ceramide species 16:0, 18:0 and 18:1 in the lipid droplet fraction (**b**), aPKCζ activation (**c**), PP2A (**d**), Akt (**e**) and serine^473^-phosphorylation of Akt relative to Akt (**f**) after ingestion of PAL (red), SAF (blue) or VCL (water, grey) at 0 min. Expression signals on immunoblots are expressed in arbitrary units (AU) after normalising against GAPDH for total and cytosolic proteins and against Na^+^/K^+^-ATPase for membrane proteins. Data are shown as means ± SEM; *n* = 16 at time point −60 min, *n* = 10 at +120 min, *n* = 6 at +240 min and +420 min. *p < 0.05 vs VCL at same time point; ^†^*p* < 0.05 for PAL vs SAF at same time point (ANOVA adjusted for repeated measures with Tukey–Kramer correction for each time point between interventions). P-basal; pre-basal

Time-dependent changes were analysed by comparing the iAUC of the respective parameters. After PAL only, concentrations of 16:0; 18:0; 18:1 ceramide species increased in the membrane compartment, compared with after VCL (~30% increase, *p* = 0.022 for PAL vs VCL; *p* = 0.078 for SAF vs VCL; Fig. 6a and ESM Table 4). Similarly, concentrations of these ceramide species in lipid droplets were ~30% and ~20% higher after PAL than after VCL or SAF (*p* = 0.017 PAL vs VCL; *p* = 0.025 PAL vs SAF; Fig. 6b). Membrane-to-cytosol translocation of aPKCζ was ~69% and ~59% higher after PAL than after VCL (*p* = 0.013) or SAF (*p* = 0.035; Fig. 6c), respectively. In addition, PAL induced a ~35% higher myocellular PP2A expression, when compared with VCL (*p* = 0.031) or SAF (*p* = 0.039; Fig. 6d). Only after PAL, serine^473^-phosphorylation of Akt was reduced by 40% and 36% compared with VCL (*p* = 0.022) or SAF (*p* = 0.034; Fig. 6e,f), respectively.

### PAL and SAF do not acutely affect mitochondrial oxidative capacity

During the basal period, SAF and PAL, compared with VCL ingestion, had no effect on total muscle CSA, maximum coupled and uncoupled mitochondrial oxidative capacity from succinate and carbonyl cyanide *p*-trifluoro-methoxyphenyl hydrazine (FCCP) normalised to CSA, β-oxidation from octanoyl-carnitine related to CSA, H_2_O_2_ emission normalised to CSA and leak control ratio, which reflects increased proton leak across the mitochondrial membrane (ESM Fig. 4a–f).

### PAL and SAF do not acutely affect biomarkers of inflammation

Variables of inflammation in the circulation (IL-6, TNF-α, cortisol) and in skeletal muscle (c-JNK, phosphorylated JNK) did not differ between interventions during basal and clamp periods (ESM Table 5).

## Discussion

This study shows that a single lipid load, regardless of its FA composition, causes whole-body and hepatic insulin resistance. Palmitate-rich lipid ingestion was associated with activation of the myocellular DAG–nPKC pathway, leading to inhibitory serine^1101^-phosphorylation of IRS-1, already during basal insulinaemia. Of note, PAL also increased myocellular ceramide content, which may contribute to skeletal muscle insulin resistance via activation of aPKCζ and PP2A and inhibition of Akt. Furthermore, neither palmitate- nor oleate-containing lipid loads acutely affected mitochondrial oxidative capacity or inflammatory pathways in human skeletal muscle.

Both SAF (enriched in oleate) and PAL (enriched in palmitate and oleate) decreased whole-body insulin sensitivity under hyperinsulinaemic conditions. Compared with SAF, PAL had a somewhat more pronounced inhibitory effect on insulin sensitivity during the clamp test, with reduction in insulin-stimulated NOXGD as well as glucose disposal during the basal period of fasting insulinaemia, as seen in most [5, 11] but not all human studies [12]. Of note, lipid loading may cause substantial substrate competition, particularly in the absence of insulin stimulation. In the present study, SAF and PAL did not seem to affect the LOX/GOX ratio during the basal period and LOX was only nominally higher under clamp conditions. Taken together, both lipid interventions markedly decreased insulin-stimulated whole-body glucose disposal, at least partly due to NOXGD, which mainly reflects skeletal muscle insulin resistance.

So far, most controlled human studies investigating lipid-induced insulin resistance have used i.v. or oral mixed lipid challenges without distinguishing between the degrees of FA saturation [19]. The present study now extends these previous results [5, 6, 19, 20] in that both single lipid meals, but especially PAL already during the basal period, acutely increased membrane C18-containing DAG species, followed by nPKCθ translocation, serine^1101^-phosphorylation of IRS-1 and whole-body insulin resistance. This sequence of events has been also demonstrated for i.v. infusion of lipids and supports the causal relationship observed in in vitro studies [21]. The subsequent decrease in nPKCθ during the clamp could be due to insulin-dependent lowering of intracellular FA-CoA levels, which may synergistically activate nPKCs [22]. Later in the course of study, DAG species also accumulated in the lipid droplet compartment, likely reflecting elevated triacylglycerol synthesis. Of note, the present study did not further analyse the C-18-containing DAG for its 1,2 stereoisomers, which are the bioactive mediators of insulin resistance [1, 20]. In line, previous studies indicate that palmitate exposure can induce higher myocellular DAG production than oleate [23, 24], associated with lower insulin-stimulated myocellular glucose uptake [25]. Furthermore, we observed increased nPKCɛ translocation after PAL but not SAF. Previous studies have linked lipid-mediated insulin resistance mainly to nPKCθ activation in rodents [26] and humans [5, 6]. However, higher skeletal muscle nPKCɛ activity was demonstrated in obesity or type 2 diabetes, compared with lean humans, as well [20].

Interestingly, only the single palmitate-rich lipid ingestion but not the oleate-rich lipid ingestion also increased the myocellular ceramide content, again first in the membrane and then in lipid droplets, the cellular lipid repository [15]. This was accompanied by activation of the aPKCζ–PP2A pathway [27, 28], by which membrane-bound ceramide is known to inhibit serine^473^-phosphorylation of Akt [29]. Exclusively, PAL decreased the myocellular serine^473^-phosphorylated Akt/Akt ratio during hyperinsulinaemia, indicating decreased distal insulin signalling. In an obese mouse model, inhibition of serine palmitoyl-transferase-1, an enzyme involved in de novo synthesis of ceramides from saturated FA, led to acute improvement of muscle Akt phosphorylation and insulin sensitivity [30, 31]. Thus, the lack of a similar effect with SAF despite the observed insulin resistance could result from different lipotoxic synthesis pathways based on the degree of saturation of the ingested FA intervention [32]. Alternatively, higher total and other NEFA concentrations following PAL vs SAF may explain PAL’s more pronounced inhibitory effect on Akt.

Generally, saturated fat, particularly palmitate, has been associated with reduced whole-body insulin sensitivity [33]. A 7-day diet intervention in lean women but not men linked a palmitate-rich diet to higher myocellular ceramides with lower insulin sensitivity compared with an oleate-rich diet [34]. Of note, in cultured human myotubes, the addition of an equal amount of oleate suddenly improved palmitate-induced ceramide-mediated insulin resistance [25, 35]. This mitigating effect of oleate on lipotoxicity through palmitate-induced increase in ceramides has been previously explained by augmented mitochondrial FA metabolism [36], although oleate and palmitate compared with palmitate alone were able to increase triacylglycerol storage, thereby preventing DAG accumulation and insulin resistance at least in cultured muscle cells [23].

Our study further confirms an increase in membrane C16- to C18-ceramide species, which were acutely increased in lean and obese humans in the context of insulin resistance upon palmitate ingestion [37]. Another study found an inverse relationship between elevated myocellular ceramides and insulin sensitivity in lean men after an i.v. infusion of mixed lipids/heparin [38]. In addition, Perreault et al reported a relationship of sarcolemmal ceramides, but not DAG, with human insulin resistance [20]. Of note, other studies found neither alterations of myocellular sphingolipids nor relationships with insulin sensitivity in people who were obese and/or had type 2 diabetes [6, 39, 40]. In addition, the finding that unsaturated FA can induce insulin resistance without elevating ceramide content suggests that the ceramide-mediated impairment of distal insulin signalling may not be mandatory for lipid-induced insulin resistance [41]. However, the observation that the palmitate-induced increase in myocellular ceramides appears to aggravate acute lipid-mediated insulin resistance when compared with oleate alone supports the hypothesis of synergic inhibition of insulin signalling by different lipid mediators, at least under conditions of a single oral lipid load [42]. Furthermore, the palmitate-enriched intervention also resulted in lower EGP during basal insulinaemia, which would favour higher rates of gluconeogenesis and lipogenesis [10, 11]. During the clamp, both SAF and, more markedly, PAL decreased hepatic insulin sensitivity. This is remarkable, as this one-step clamp was not primarily designed to examine hepatic insulin action, indicating a strong stimulatory effect on EGP, which may be explained by FA-induced allosteric stimulation of gluconeogenesis and, to a minor extent, by increased glycerol uptake, serving as gluconeogenic substrate [1, 2]. In addition, incretin-mediated changes in portal insulin and glucagon concentrations may mask the effects of oral lipid intake on hepatic glucose metabolism [43]. Furthermore, higher GLP-1 concentrations may lead to increased NEFA uptake from plasma chylomicrons and suppression of FA spillover into the circulation [44]. This could explain the absent increase in NEFA despite elevated chylomicrons after SAF vs PAL seen in the current study. Previous acute high-fat diet intervention studies demonstrated similar [10, 11] or no effects in humans [5, 45], suggesting that the different results are not due to lipid composition but rather due to study design or total energy intake.

Interestingly, the present study failed to detect changes in circulating inflammatory cytokines, cortisol or classical cellular inflammatory pathways in line with previous studies on acute effects of saturated [11] and monounsaturated FAs in humans [5, 10]. This underlines that the known increase of low-grade inflammation occurring upon high-fat diet [34] is not involved in the acute initiation of muscle insulin resistance but rather results over time from secondary alterations, likely arising from adipose tissue dysfunction [1]. Likewise, the present study found no acute effects of PAL or SAF on muscle mitochondrial enzyme activity, oxidative capacity or H_2_O_2_ emission during the basal period, in line with previous studies [46], supporting the concept that lipotoxic signalling is the primary event of lipid-induced muscle insulin resistance, regardless of the lipid composition. Nevertheless, excessive mitochondrial H_2_O_2_ production has been linked to insulin resistance in a high-fat diet model in rodents and humans [47], indicating effects on mitochondrial function operating during long-term lipid overfeeding.

The present study benefits from the serial biopsies allowing for real-time monitoring and analyses of the sequence of cellular events in human skeletal muscle, although by design it cannot provide a definite mechanistic proof. Limitations include the use of pure oils, rather than the consumption of mixed meals. In addition, using water as control will lead to specific metabolic and endocrine conditions (e. g. substrate competition, lipolysis with endogenous NEFA spill-out [48] and low level postprandial incretins). The lipid interventions were designed to be isoenergetic (~3012 kJ) so that differences in effects are unlikely to be due to energy intake. Also, the fact that no strict monitoring of diet and exercise was feasible on the days before the interventions might have influenced the acute lipid overload response. Moreover, the results of this short-term study in healthy humans cannot be necessarily extrapolated to chronic lipid oversupply or common insulin resistance of obesity and type 2 diabetes. Nevertheless, our approach represents an accepted compromise between optimal experimental and near-physiological conditions to allow for assessing acute metabolic changes [10–12].

In conclusion, this short-term study reveals that raising plasma oleate with and without palmitate concentrations is associated with activation of the myocellular DAG–nPKC pathway and muscle insulin resistance. The acute increase in plasma palmitate also favours ceramide accumulation and aPKCζ–PP2A activation, which can further aggravate muscle insulin resistance. The possible role of lipid interactions, such as modulation by oleate of the palmitate-induced inhibitory signalling events, remains to be investigated. All these effects seem to occur in the absence of changes in mitochondrial capacity or inflammatory pathways, underlining the relevance of lipotoxic pathways as therapeutic targets to prevent and reverse muscle insulin resistance in humans. Finally, further studies are required to prove the effects of diets containing different lipids in healthy and insulin-resistant humans.

## Supplementary information

ESM 1 (PDF 751 kb)

## Data Availability

All data generated or analysed during this study are included in the published article (and its online supplementary files). The files are available from the corresponding author upon reasonable request.

## Abbreviations

ALT: Alanine aminotransferase
AST: Aspartate aminotransferase
aPKC: Atypical protein kinase C
BSA: Body surface area
BW: Body weight
CSA: Citrate synthase activity
DAG: Diacylglycerol
DDZ: German Diabetes Center
EGP: Endogenous glucose production
FA: Fatty acid
FAME: FA methyl ester
GIP: Gastric inhibitory polypeptide
GLP-1: Glucagon-like peptide-1
JNK: Jun N-terminal kinase
GOX: Glucose oxidation
iAUC: Incremental AUC
LOX: Lipid oxidation
NOXGD: Non-oxidative glucose disposal
nPKC: Novel protein kinase C
PAL: Palm oil
PP2A: Protein phosphatase 2A
*R*_d_: Rate of glucose disposal
REE: Resting energy expenditure
SAF: Safflower oil
VCL: Vehicle
