# Supplementary material for: Dietary palmitate and oleate differently modulate insulin sensitivity in human skeletal muscle
Source: Diabetologia. 2021 Oct 26;65(2):301–14. doi: 10.1007/s00125-021-05596-z (PMC8741704; doi:10.1007/s00125-021-05596-z)
Supplement: Supplementary file 1 — (PDF 751 kb) [file 125_2021_5596_MOESM1_ESM.pdf]

Electronic Supplementary Material (Dietary palmitate and oleate differently modulate insulin sensitivity in human skeletal muscle)

ESM Table 1 Participants‘ anthropometric and blood parameters, given as means ± SD or median (interquartile range).

Parameter	Total	Men	Women
**n**	16	10	6
**Age (years)**	25 ±4	27±9	23±5
**Body mass index (kg/m^2)**	23±2	24±1	22±3
**Waist circumference (cm)**	78±5	81±5	75±5
**Body fat (%)**	22±6	18±4	26±5
**Systolic blood pressure (mmHg)**	124±12	119±5	121±10
**Diastolic blood pressure (mmHg)**	77±8	77±9	82±7
**Fasting blood glucose (mmol/l)**	4.3±0.3	3.9±0.4	4.5±0.3
**2-h OGTT blood glucose (mmol/l)**	4.3±1.0	4.1±0.6	4.6±0.8
**M-value (mg kg^{-1} min^{-1})**	8 (1)	8 (1)	8 (1)
**Fasting plasma NEFA (µmol/l)**	543 (225)	573 (275)	553 (259)
**Fasting plasma triacylglycerol (mmol/l)**	4.2 (3.3)	3.9 (3.3)	3.9 (3.1)

OGTT, oral glucose tolerance test; NEFA, non-esterified fatty acids

ESM Table 2 Plasma non-esterified fatty acids (NEFA), given as means ± SD, during the pre-basal (-120 min – 0 min) and basal periods (0 min - +360 min) in healthy humans after palm oil (PAL), safflower oil (SAF) or vehicle (water, VCL) ingestion at 0 min.

NEFA (μmol/l)	Intervention	Time		
		+0 min	+180 min	+360 min
**Myristic acid (14:0)**	VCL	3±1	2±1	1±0.4
	SAF	2±0.1	1±0.3	1±0.1
	PAL	7±1	6±1*	7±1*
**Palmitoleic acid (16:1)**	VCL	7±1	5±0.3	6±3
	SAF	2±0.2	1±0.4	1±0.6
	PAL	8±1	6±1	4±2*
**Stearic acid (18:0)**	VCL	8±1	6±1	17±2
	SAF	10±1	8±1	12±2
	PAL	5±9	15±7††*	27±4

n = 5; *p < 0.05 vs. VCL of the same time point; ††p < 0.01 vs. 0 min of the same group; ANOVA adjusted for repeated measures with Tukey-Kramer correction.

ESM Table 3 List of parameters during steady-state clamp period (+460 min – +480 min), given as means ± SD, in healthy humans after palm oil (PAL), safflower oil (SAF) or vehicle (water, VCL) ingestion at 0 min.

**Parameter**	**Group**	**Time**		
		**+460 min**	**+470 min**	**+480 min**
**GIR (ml kg^{-1} h^{-1})**	VCL	1.7±0.7	1.7±0.8	1.8±0.9
	SAF	1.5±0.6	1.35±0.5	1.33±0.6
	PAL	1.3±0.5	1.3±0.4	1.3±0.4
**APE [$^2H_2$]glucose (%)**	VCL	2.3±0.2	2.5±0.1	2.4±0.1
	SAF	2.7±0.1	2.7±0.1	2.8±0.1
	PAL	2.4±0.2	2.4±0.1	2.5±0.2

n = 16; ANOVA adjusted for repeated measures with Tukey-Kramer correction. GIR, glucose infusion rate; APE, atom percent enrichment

ESM Table 4 Skeletal muscle total diacylglycerols (DAG) and ceramides, all given as means ± SD, in subcellular compartments during the pre-basal (-120 min – 0 min), basal (0 min - +360 min) and clamp periods (+360 min - +480 min) in healthy humans after palm oil (PAL), safflower oil (SAF) or vehicle (water, VCL) ingestion at 0 min.

**Lipid (nmol/g)**	**Intervention**	**Time**			
		**-60 min**	**+120 min**	**+240 min**	**+420 min**
**DAG cytosol**	VCL	0.57±0.22	0.59±0.36	0.32±0.21	0.80±0.39
	SAF	0.45±0.23	0.68±0.44	0.47±0.19	0.82±0.29
	PAL	0.54±0.32	0.66±0.39	0.45±0.21	0.86±0.23
**DAG membrane**	VCL	6.06±2.86	5.93±1.66	6.36±2.94	6.44±2.26
	SAF	6.33±2.16	6.52±2.08	8.61±2.85	10.06±3.08
	PAL	6.28±2.70	6.93±2.94	9.93±3.31	11.22±2.62
**DAG lipid droplet**	VCL	0.65±0.33		0.81±0.60	0.93±0.42
	SAF	0.74±0.25		0.91±0.53	1.06±0.30
	PAL	0.74±0.39		0.83±0.20	1.53±0.44
**Ceramide cytosol**	VCL	0.17±0.06	0.17±0.08	0.19±0.09	0.17±0.07
	SAF	0.20±0.11	0.24±0.13	0.22±0.08	0.26±0.13
	PAL	0.19±0.11	0.21±0.13	0.28±0.09	0.31±0.11
**Ceramide membrane**	VCL	1.43±0.35	1.58±0.28	1.59±0.72	1.68±0.51
	SAF	1.70±0.38	1.71±0.39	1.60±0.24	1.63±0.41
	PAL	1.44±0.45	1.50±0.33	1.91±0.39*	1.60±0.44
**Ceramide lipid droplet**	VCL	0.03±0.01		0.02±0.02	0.04±0.01
	SAF	0.04±0.01		0.03±0.02	0.05±0.02
	PAL	0.03±0.01		0.03±0.02	0.04±0.02

-60 min: n = 16; +120 min: n = 10; +240 min and +420 min n = 6; +120 min: DAG and ceramide concentration in the lipid droplet compartment was not determined; *$p < 0.05$ vs. VCL of same time point; ANOVA adjusted for repeated measures with Tukey-Kramer correction revealed no significant differences.

ESM Table 5 Biomarkers of inflammation, all given as means ± SD, in subcellular compartments during the pre-basal (-120 min – 0 min), basal (0 min - +360 min) and clamp periods (+360 min - +480 min) in healthy humans after palm oil (PAL), safflower oil (SAF) or vehicle (water, VCL) ingestion at 0 min.

Variable	Intervention	Time				
		**-120 min**	**+120 min**	**+240 min**	**+360 min**	**+480 min**
**Plasma IL-6 (pg/ml)**	VCL	0.8±0.4	1.8±0.7	3.2±0.7	3.4±0.7	4.1±1.1
	SAF	1.3±1.0	1.7±1.0	3.1±2.7	3.3±2.0	4.3±4.1
	PAL	0.8±0.1	1.6±1.0	2.6±1.9	3.3±2.0	4.9±4.1
**Plasma TNF-α (pg/ml)**	VCL	0.8±0.2	0.7±0.1	0.6±0.1	0.6±0.1	0.6±0.1
	SAF	0.7±0.2	0.8±0.3	0.7±0.2	0.7±0.2	0.6±0.2
	PAL	0.7±0.2	0.7±0.3	0.7±0.2	0.6±0.2	0.6±0.2
**Plasma cortisol (nmol/l)**	VCL	366±8	228±77	259±49	264±82	311±52
	SAF	535±57	369±41	333±104	284±33	270±60
	PAL	471±52	361±46	309±113	286±60	273±118
**Muscle JNK total (AU)**	VCL	2.1±1.3	1.7±0.9	2.1±0.9	1.4±0.6	1.6±0.8
	SAF	2.7±1.5	1.3±0.5	2.3±0.8	1.4±0.5	1.9±0.6
	PAL	2.5±1.2	1.4±0.8	1.7±0.7	1.3±0.5	1.4±0.5
**Muscle pJNK total (AU)**	VCL	1.3±0.6	1.4±0.7	2.0±0.9	0.9±0.2	0.8±0.4
	SAF	1.7±1.4	1.0±0.5	1.4±0.6	0.9±0.2	0.9±0.6
	PAL	1.3±0.8	1.1±0.8	2.2±0.4	0.7±0.2	0.7±0.2

n = 14-16; ANOVA adjusted for repeated measures with Tukey-Kramer correction revealed no significant differences. IL-6, Interleukin 6; TNF-α, tumor necrosis factor; JNK, c-Jun N-terminal kinases; p-JNK, phosphorylated c-Jun N-terminal kinases; AU, arbitrary units

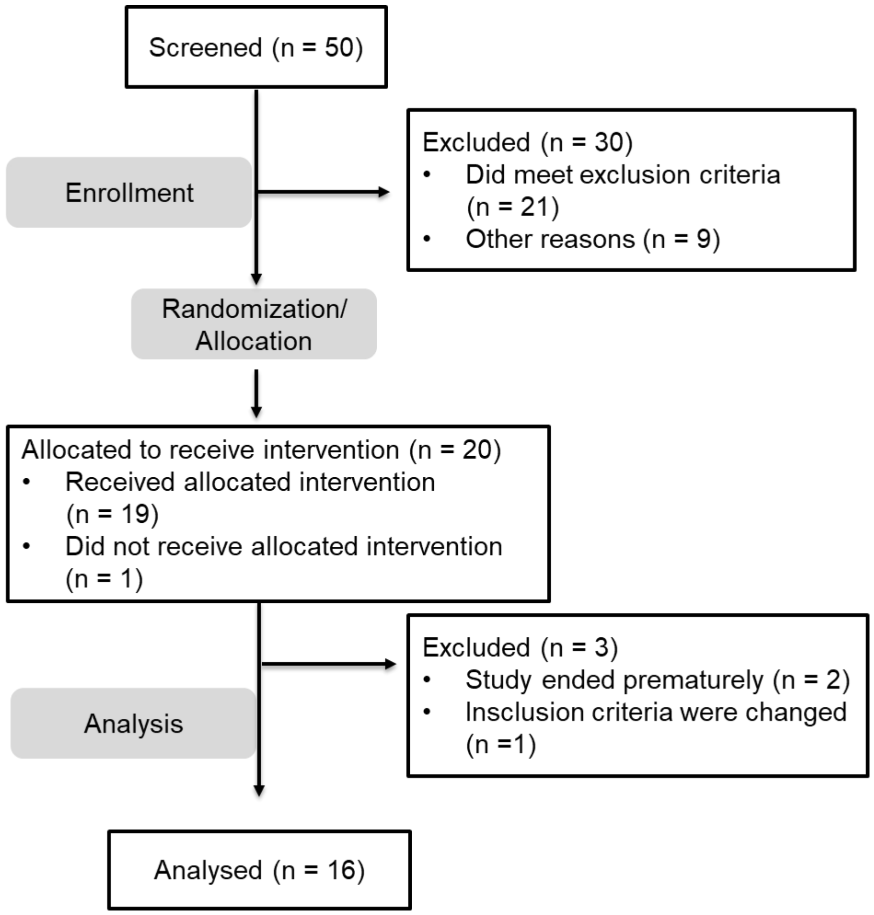

ESM Fig. 1 CONSORT flow diagram. Fifty volunteers underwent screening, which included medical history, anthropometric and bioimpedance analyses as well as a 75-g oral glucose tolerance test (OGTT). Twenty-one persons did not meet the inclusion criteria and 9 were excluded for other reasons. Of the remaining 20 participants, all but one receive the interventions. During the study, two volunteers were excluded prematurely and another one had to be excluded after a change of the inclusion criteria.

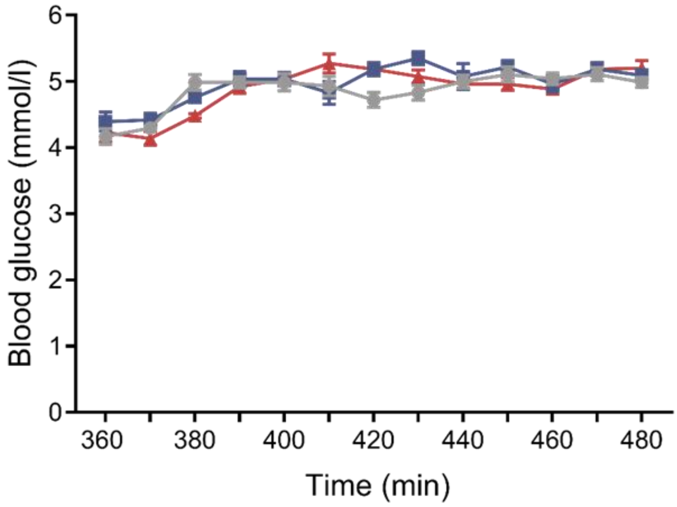

ESM Fig. 2 Changes in circulating blood glucose during the clamp period in healthy humans. Blood glucose is presented during a hyperinsulinemic-euglycemic clamp test (+360 min – +480 min) with D-[6,6-$^{2}H_{2}$]glucose administration performed according to the "hot" glucose infusion (hot-GINF) protocol in healthy humans after palm oil (PAL, red), safflower oil (SAF, blue) or vehicle (water, VCL, grey) ingestion at 0 min. Data are shown as means ± SEM; *n* = 16 (ANOVA adjusted for repeated measures with Tukey-Kramer correction revealed no significant differences).

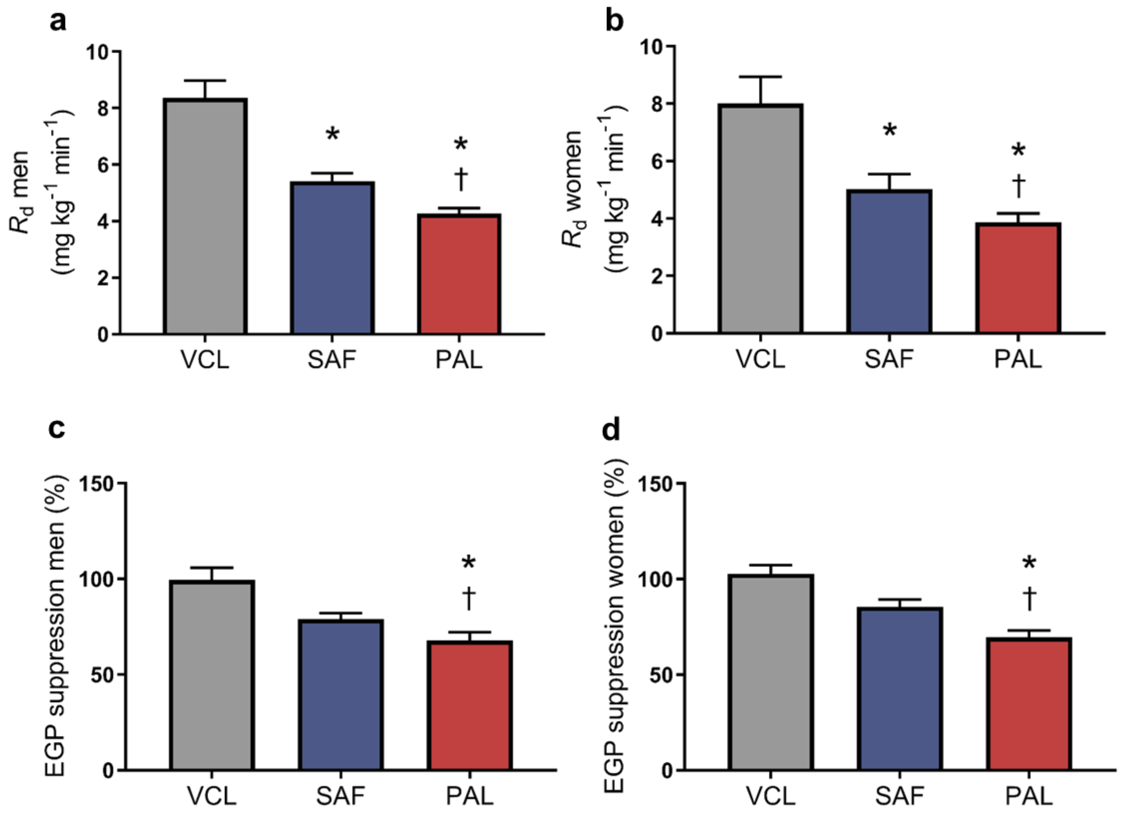

ESM Fig. 3 Differences in sex of whole-body glucose disposal (R_d) and suppression of endogenous glucose production (EGP) during clamp period in healthy humans. During the steady state clamp period (+460 min - +480 min), insulin-stimulated glucose disappearance (R_d) for men (**a**) as well as R_d for women (**b**) and suppression of endogenous glucose production (EGP) for men (**c**) and EGP suppression for women (**d**) are presented after palm oil (PAL, red), safflower oil (SAF, blue) or vehicle (water, VCL, grey) ingestion at 0 min. Data are shown as means ± SEM; n = 16. *$p<0.05$ vs. VCL; †$p<0.05$ PAL vs. SAF (ANOVA adjusted for repeated measures with Tukey-Kramer correction revealed no significant differences).

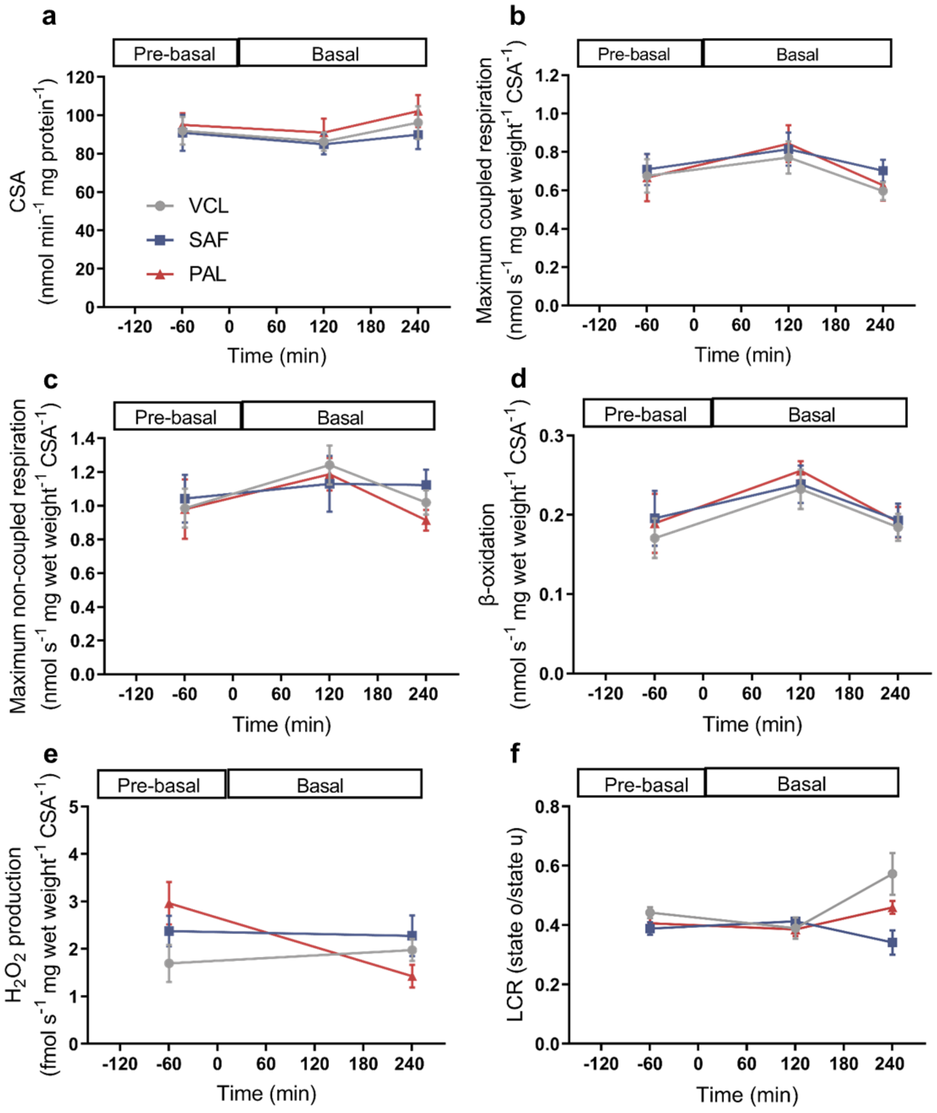

ESM Fig. 4 Skeletal muscle energy metabolism in healthy humans. Citrate synthase activity (CSA, **a**), maximum coupled mitochondrial oxidative respiration normalized to CSA (O_2 flux measured in freshly isolated skeletal muscle mitochondria after applying succinate; **b**), maximum non-coupled mitochondrial oxidative respiration normalized to CSA (O_2 flux measured in freshly isolated skeletal muscle mitochondria after applying carbonyl cyanide 4-trifluoromethoxy-phenylhydrazone, FCCP; **c**), ß-oxidation normalized to CSA (O_2 flux measured in freshly isolated skeletal muscle mitochondria after applying octanoyl-carnitine; **d**), H_2O_2 production normalized to CSA (measured in freshly isolated skeletal muscle mitochondria after applying oligomycin under state 4 conditions; **e**) and leak control ratio (calculated from the ratio of leak state in the presence of oligomycin (state o) and electron transport capacity at optimum concentration of FCCP (state u) (LCR, **f**) during the pre-basal (-60 min) and basal periods (+120 min and +240 min) after palm oil (PAL, red), safflower oil (SAF, blue) or vehicle (water, VCL, grey) ingestion at 0 min. Data are shown as means ± SEM; n = 16 at time point -60 min; n = 10 at +120 min, n = 6 at +240 min (ANOVA adjusted for repeated measures with Tukey-Kramer correction revealed no significant differences).
